# MASQ: Toward clinical quantification of disease-associated alternative splicing

**DOI:** 10.1016/j.omtn.2026.103021

**Published:** 2026-07-31

**Authors:** Yvonne Xinyi Lim

**Affiliations:** 1 Department of Periodontics and Oral Medicine, University of Michigan School of Dentistry, 1011 N. University Ave, Ann Arbor, MI, USA; 2 Rogel Cancer Center, University of Michigan, 1500 E Medical Center Dr, Ann Arbor, MI, USA

## Main text

Alternative splicing (AS) is a post-transcriptional regulatory mechanism that enables individual genes to generate multiple transcript and protein isoforms with distinct structures and functions. It is estimated that AS occurs in approximately 95% of human multi-exon genes,^1^ and that each human protein-coding pre-mRNA may generate an average of 5–7 spliced isoforms.^2^ Dysregulated splicing can alter essential biological processes and has been implicated in multiple diseases, including cancer, neurodegenerative disease, developmental and immune disorder. Thus, splicing dysregulation may be used as disease biomarkers and therapeutic targets.

The translational relevance of splicing has increased substantially with the emergence of antisense splice-switching oligonucleotides (SSOs) and related RNA-targeted therapies. In recent years, the approval of two SSO therapies by the US Food and Drug Administration, nusinersen for spinal muscular atrophy^3^ and eteplirsen for Duchenne muscular dystrophy,^4^ demonstrates the therapeutic potential of targeting disease-associated splicing defects. The flexibility of SSO design creates opportunities to correct patient-specific splicing defects in rare genetic disorders, enabling individualized and variant-specific therapeutic strategies.^5^^,^^6^ These advances highlight the need for reliable methods to quantify predefined splice events in patient samples. Accurate splicing quantification is key for biomarker discovery, patient stratification, pharmacodynamic monitoring of splice-switching therapies, and assessment of therapeutic response in clinical trials.

**Figure 1:**
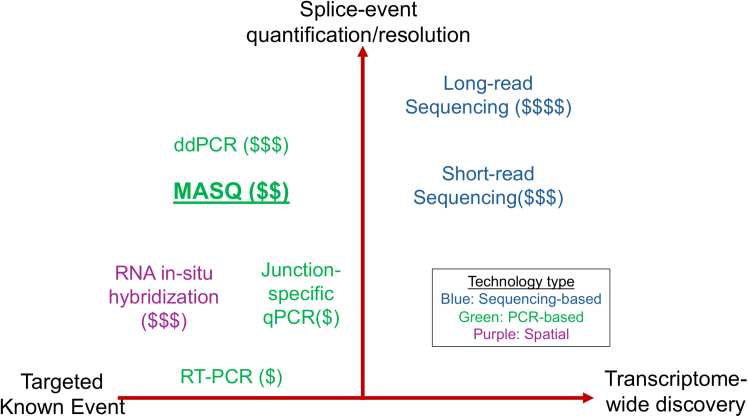
Technology landscape for alternative splicing quantification Current approaches differ in discovery breadth, splice-event resolution, cost, and clinical scalability. Short-read RNA-seq enables transcriptome-wide discovery but often requires computational isoform inference, whereas long-read sequencing provides higher full-length isoform resolution but are very costly. Targeted methods, including RT-PCR, qPCR, ddPCR, RNA *in situ* hybridization, and MASQ, require prior knowledge of the splice event. Among these, MASQ uses standard qPCR infrastructure to provide scalable exon-inclusion quantification. Dollar signs indicate relative cost and complexity.

Despite this growing need, current approaches for AS quantification have substantial limitations (Figure 1).^7^ Short-read RNA sequencing is useful for transcriptome-wide discovery of splicing events. However, because it sequences only short fragments of transcripts (<300 base pairs), resolution of specific isoforms is low and often depends on computational inference. This is especially challenging for genes with complex isoform patterns, rare isoforms, or repetitive regions. Long-read sequencing can more directly resolve the transcript structures of full-length and spliced isoforms by capturing longer RNA or cDNA molecules, but its clinical scalability is limited by cost, RNA-quality requirements and the depth needed to detect rare isoforms. Targeted PCR-based methods, including junction-specific qPCR and droplet digital PCR, can sensitively detect predefined splice junctions or isoforms, but they may be limited by amplification bias, restricted multiplexing, and difficulty resolving complex isoform patterns. RNA *in situ* hybridization approaches provide valuable spatial context in intact tissues but require careful probe design and validation, which may be challenging for highly similar isoforms. Together, these limitations highlight the need for scalable, accurate, and clinically practical methods to quantify predefined disease-relevant splice events in patient specimens.

In this study,^8^ published in the June issue of *Molecular Therapy Nucleic Acids*, Jeoug and colleagues developed multiplex alternative splicing quantification (MASQ), a platform for quantitative AS analysis in clinical specimens. MASQ adapts conventional probe-based qPCR by using dual TaqMan probes in a single reaction: one probe detects a predefined exon, while a second probe detects a constitutive exon for internal normalization. This design enables MASQ to estimate the relative inclusion of a predefined exon. Rather than serving as a discovery platform, MASQ is positioned as a practical, scalable, and quantitative assay for known splice events.

The authors used AS of *PBRM1* exon 27 as a model system.^8^ *PBRM1* encodes a component of the PBAF chromatin remodeling complex. Inclusion of exon 27 produces a full-length PBRM1 isoform that promotes PBAF complex recruitment to the PD-L1 promoter, increasing PD-L1 expression and contributing to resistance to PD-1 blockade.^9^ In contrast, exon 27 exclusion disrupts this function and has been associated with increased sensitivity to immune checkpoint therapy.^9^ This biological context makes *PBRM1* exon 27 an attractive candidate splice event for biomarker development.

Jeong et al. validated MASQ using plasmid standards, cancer cell lines, and CRISPR-engineered cells lacking *PBRM1* exon 27.^8^ This platform was also applied to clinical tissue specimens from patients with uterine corpus endometrial carcinoma, where MASQ detected higher *PBRM1* exon 27 inclusion in cancer tissues than in normal endometrium. Although preliminary, these findings support the potential utility of MASQ for analysis of patient-derived material and demonstrate how MASQ could be scaled to larger cohorts to test whether *PBRM1* exon 27 inclusion could serve as a predictive biomarker for immunotherapy response. To demonstrate broader applicability, the authors adapted the platform to quantify *HTRA2* exon 7 inclusion.^8^ In this second model, MASQ monitored SSO-induced exon 7 skipping over time, extending the platform’s potential beyond biomarker studies to pharmacodynamic monitoring of RNA-targeted therapies.

The main strength of MASQ is that it fills a practical gap between transcriptome-wide splicing discovery and clinically deployable splicing quantification. RNA sequencing (RNA-seq) and long-read sequencing are powerful for identifying and resolving splice isoforms across the transcriptome, but they may be less practical for routine measurement of selected splice events across large numbers of patient specimens. In contrast, MASQ leverages standard qPCR infrastructure while introducing a dual-probe multiplex design that simultaneously detects an alternative exon and a constitutive reference exon in a single reaction (Figure 1). By converting these Ct values into a calibrated estimate of exon inclusion, MASQ reduces variability related to RNA input, reverse-transcription efficiency, and separate-reaction normalization while retaining the accessibility of conventional qPCR platforms. By measuring the alternative and constitutive exon signals in the same reaction, MASQ reduces the need for separate normalization reactions and avoids reliance on gel-based band intensity. As a result, the platform is relatively inexpensive, scalable, and well suited to translational studies requiring reproducible analysis of multiple samples. MASQ should be viewed not as a replacement for sequencing, but as a complementary downstream tool: sequencing can identify candidate disease-relevant splice events, whereas MASQ can validate and monitor selected events at scale.

Nevertheless, several limitations remain. First, MASQ is a targeted assay and therefore requires prior knowledge of the splice event being measured. Second, because MASQ quantifies inclusion of a specified exon relative to a constitutive exon, it does not necessarily define full-length transcript identity. Third, accurate exon-inclusion estimates require rigorous validation of primer efficiency, probe specificity, assay linearity, and dynamic range for each target. Clinical implementation will also require attention to pre-analytical variables. Patient specimens often contain mixed cell populations, variable tumor purity, stromal or immune-cell contamination, and differences in RNA quality caused by tissue handling, storage, or fixation. Future studies should therefore evaluate MASQ performance in formalin-fixed paraffin-embedded (FFPE) tissues, low-input samples, and multi-site clinical-trial specimens. Inter-laboratory reproducibility and standardized exon-inclusion thresholds will also be essential if MASQ-based assays are to support clinical decision-making.

The current study does not yet establish *PBRM1* exon 27 inclusion as a clinically validated predictive biomarker for immune checkpoint inhibitor response. Prospective clinical studies will be needed to determine whether MASQ-derived *PBRM1* exon 27 inclusion predicts treatment outcomes in patients. Larger independent cohorts, ideally linked to immunotherapy response data, will be necessary to define clinically meaningful cutoffs and determine whether this splice event adds predictive value beyond existing biomarkers.

In summary, MASQ represents an accessible platform to quantify targeted AS events. Its application to *PBRM1* highlights its potential for biomarker development, while its adaptation to *HTRA2* demonstrates broader utility for monitoring splice-switching interventions. More broadly, this study underscores an important translational priority: the development of accurate, reproducible, and clinically scalable tools for splicing quantification. As disease-relevant splice isoforms become increasingly important as biomarkers, therapeutic targets, and pharmacodynamic readouts, greater effort should be directed toward methods that can reliably measure defined splicing events in patient specimens and clinical trials.

## Acknowledgments

No funding is listed for this commentary.

## Declaration of interests

The author is listed on a provisional patent application related to RNA therapeutics, which is not discussed in the present work.
